# Spatial and seasonal groundwater quality assessment for drinking suitability using index and machine learning approach

**DOI:** 10.1016/j.heliyon.2024.e30362

**Published:** 2024-04-27

**Authors:** Kibru Gedam Berhanu, Tarun Kumar Lohani, Samuel Dagalo Hatiye

**Affiliations:** a Arba Minch Water Technology Institute, Faculty of Water Resources and Irrigation Engineering, Arba Minch University, Arba Minch, Ethiopia; b Arba Minch Water Technology Institute, Faculty of Hydraulic and Water Resources Engineering, Arba Minch University, Arba Minch, Ethiopia

**Keywords:** Drinking suitability, Drinking water quality index, Hydrogeochemical facies, Physicochemical characteristics, Support vector machine, Dry and wet seasons

## Abstract

Spatial and seasonal evaluation and monitoring of groundwater (GW) quality is essential for the sustainable management of this priceless resource and the provision of safe drinking water. Nevertheless, GW quality appraisal was not given due attention in the current study area (flat terrain part of the Tana sub-basin). This study sought to assess the seasonal and regional physicochemical GW quality parameters for drinking appropriateness using the drinking water quality index (DWQI) and support vector machine (SVM). The main cations in both the dry and wet study seasons were, in decreasing order, Na^+^, Ca^2+^, Mg^2+^, K^+,^ and Fe^2+^, according to the results. Conversely, the main anions were HCO^3−^, CO_3_^2−^, Cl^−^ or NO^3−^, SO_4_^2−^ and PO_4_^3−^, ordered from higher to lower. During the two research seasons, Ca–HCO_3_ and Na–HCO_3_ were the predominant water types based on Piper diagram results. Reverse ion exchange and evaporation were the principal hydrogeochemical processes that control the hydrogeochemistry identified by Durov and Gibbs diagrams, respectively. Excellent GW quality class for drinking was demonstrated by the majority of geographical and seasonal DWQI readings over the two seasons. Nevertheless, during the rainy season, there was a noticeable decline in the GW quality condition around the northern shores of Lake Tana. Therefore, it is advised to implement comprehensive GW quality protection measures and improve system management to mitigate pollution to reduce health hazards in the examined region.

## 1 Introduction

Groundwater (GW) plays a fundamental role in satisfying various demands for over fifty percent of the population across the world [1,2]. Groundwater is indispensable for life existence and healthy lifestyle, economic growth, and environmental sustainability of developing countries in particular and the world in general [3,4]. GW is more imperative than surface water owing to its relative purity, reliability and productivity, cost-effectiveness to extract and use, less vulnerability to anthropogenic influence, and climate change [3,5,6]. Nevertheless, GW resources have threatened the quality and quantity and it has become a major worry in many countries around the globe attributed to population growth and increased demands, fast-growing urbanization, intensified agricultural activities, and climate changes [7,8].

GW pollution, in Ethiopia, has been a concern traced back many decades. It was identified that the total dissolved solids (TDS), fluoride, and nitrate are the major pollutants that exceed the World Health Organization (WHO) maximum standard limit in sedimentary formations and the Rift zones, particularly in urban areas [9,10]. This investigation stepped back decades ago [9,10], pointed out that how the present groundwater resource could be vulnerable to significant pollution risks due to the continued population growth, agricultural expansions and the concomitant increased demands [11]. Recent studies [[12], [13], [14], [15], [16], [17]] confirmed the fact that groundwater contamination peril is in existence.

Particularly, the flat terrain of the Tana sub-basin (the present study area) in which GW resource is extracted mainly from tertiary and quaternary volcanic aquifers including quaternary alluvial and lacustrine deposits [18] faced quality problems. It was confirmed that poor-quality groundwater was pumped from some deep boreholes in the study region [19]. Scholars [13] tried to show the Tana sub-basin's GW vulnerability to pollution by applying the overlay method. They employed the “DRASTIC” technique that includes seven thematic layers viz. water table depth, recharge, aquifer permeability, soil permeability, topography, vadose zone impact, and aquifers conductivity. Their study result revealed that GW around Lake Tana was highly vulnerable to pollution. A very similar recent study [20], which was conducted in the northern part of the Tana sub-basin, applying a modified “DRASTIC” method indicated the higher vulnerability of GW especially around urban regions. These researchers used land use/cover thematic layers besides the traditionally employed thematic layers and fifteen groundwater samples for validation. They found that nitrate concentration was the major pollutant of the region.

Researchers [21] investigated the nitrate concentration in GW by collecting 213 drinking water samples from springs and wells in the study period from December 2013–June 2014 over the study area covering 4880 km^2^, which includes the eastern slice of the present study area. The spatial variations of the nitrate levels were assessed. Finally, the researchers speculated that the nitrate levels in GW were high in agricultural areas and may exacerbate when fertilizer utilization is escalated in the study region. Other researchers [22] also conducted GW quality appraisal by water quality index and GIS approach from thirty GW samples' physicochemical laboratory analysis in the northern portion of the Tana sub-basin. They found that many shallow wells near Lake Tana were polluted and unfit for drinking purposes. As previously, mentioned, the earlier studies focused mainly on either showing GW vulnerability to pollution or one-time GW samples for physicochemical analysis. To the best of the authors’ knowledge, there were no other studies until the commencement of the present study in the region that can provide essential information about the actual groundwater quality conditions variation spatially and seasonally. To fill this gap, therefore, the spatial and seasonal assessment of GW quality for drinking suitability by applying a simple and understandable approach is crucial.

Groundwater in the flat terrain part of the Tana sub-basin is one of the largest potential reserves in Ethiopia and is used for water supply to over three million populations [[23], [24], [25], [26]]. To sustainably preserve and manage the quality of this huge and precious resource for sustainable drinking and other purposes, a comprehensive GW quality study with actual physicochemical parameters is compulsory [27]. Hence, the identification of major hydrogeochemical facies, geochemical controlling mechanisms, and drinking suitability evaluation are essential for a complete GW quality evaluation [27,28]. Hydrogeochemical facies are imperative for deciphering the hydrogeochemical process and for GW quality and its trend appraisal [29,30]. Groundwater quality and its geochemical characteristics depend on dissolution, mineral solubility, ion exchange process, and recharge sources [27,[29], [30], [31]].

To recognize the overall water quality status spatially and seasonally, simple and understandable techniques and geostatistical need to be used for easy provision of decisive information to water-sector managers and decision-makers [32,33]. Therefore, classic graphical methods (Piper, Durov and Gibbs diagram), Drinking Water Quality Index (DWQI) and Support Vector Machine (SVM) interpolation technique were applied. Many scholars used the Piper diagram, Durov and/or Gibbs diagram to decipher the hydrogeochemical facies, and to characterize the hydrogeochemical process and mechanisms, respectively [27,[34], [35], [36]]. DWQI is a robust and widely used technique to recapitulate complex water quality parameters simply and understandably to classify the appropriateness of drinking water [33]. Support Vector Machine (SVM) is the emerging tool developed in the 1990s and effectively used among machine learning algorithms in several applications such as interpolations of DWQI values, prediction of water quality studies, classifications, and several other applications [[37], [38], [39]]. SVM performs best in geospatial interpolation especially when the data has no noise and outliers [40]. The objectives of this study are to (1) estimate the concentrations of dominant physicochemical GW quality parameters and assess their variations in the dry and wet seasons through standard laboratory analysis, (2) determine major water types and assess governing hydrogeochemical processes, and (3) evaluate the suitability of GW quality for drinking purposes spatially and seasonally. This study was the first of its kind GW quality research that was carried out in the hotspot area of Tana sub-basin coupling DWQI and SVM. The outcomes of this research may deliver full picture of the spatial and seasonal groundwater quality variation and status including the governing mechanisms controlling hydrochemistry to the end users, scientific communities, and practitioners.

## 2 Materials and methods

### 2.1 Study area

**Fig. 1:**
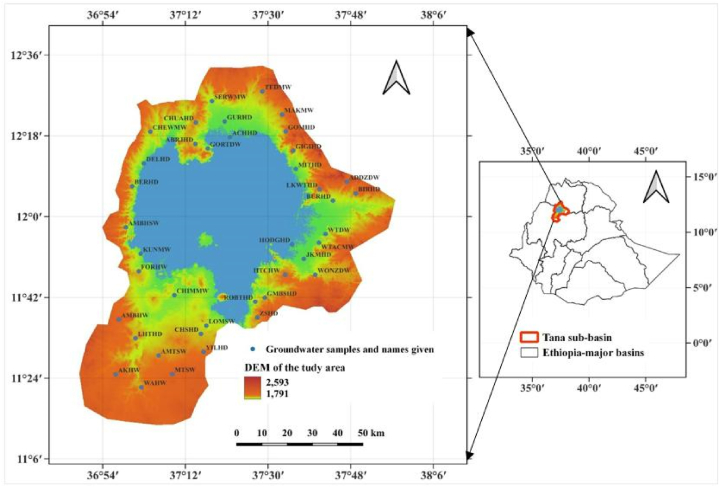
Study area and location of the groundwater samples.

The Tana sub-basin's flat terrain portion is geographically positioned in northwestern Ethiopia (Fig. 1). The DEM (digital elevation model) map of the study area shows 1791 and 2593 m minimum and maximum elevations at mean sea level, respectively (Fig. 1). The climate of this study region is governed by tropical highland monsoon, which has generally, the rainy season from June to September and the dry from October to March seasons. The study region has 1400 mm and 1329 average rainfall and potential evapotranspiration, respectively [7,41].

There are nine identified land use/cover (LULC) types (Fig. S1). The dominant LULC types are water bodies, cropland, and grassland [7]. The water body (mainly Lake Tana) covers 30 % of the total study area. Among the remaining 70 % of LULC types, cropland and grassland cover 60 % and 21 %, respectively (Fig. S1). Ten types of soils are identified and mapped (Fig. S2). Eutric vertisols, Haplic Luvisols, Chromic Luvisols, and Eutric Leptosols are the primary soil types which cover, 26.46 %, 25.91 %, 19.44 %, 15.52 %, respectively [25] (Fig. S2). Eutric cambisols cover the least area (0.01 %) among all the study area's soils.

There are ten types of lithological units including basalts related to volcanic centre (2), basalts related to volcanic centre (u), basalts related to volcanic centre (3), colluvium, Amba Aiba basalts, lacustrine deposits, alluvium, Amba Alaji Rhylite, marsh soil, and Tarmaber basalts(2) [23,25,42] (Fig. S3). Tarmaber basalts (2) and basalts related to volcanic centre (2) are the largest lithological units covering 76.55 % and 14.59 %, respectively [23]. The least lithological unit (covers 0.001 % of the study area) is Amba Alaji rhyolite. The main aquifer types found in the Tana sub-basin including the present study area are tertiary volcanic aquifer (comprises, Amba Aiba basalts, Amba Alaji Rhyolite and Tarmaber lithological unit), Quaternary volcanic aquifer (consists of different volcanic centre basalts and colluvium), and aquifers of Quaternary alluvial and lacustrine deposits [43]. These aquifers are mostly shallow and unconfined [43,44]. The water table depths’ of these shallow aquifers range from 1 to 30 m [13,45]. Among hydrogeological units, the Quaternary volcanic aquifer situated in the southern direction of the study region (basalts related to volcanic centre (2)) has the highest groundwater potential [44].

Generally, the study area is rich in a huge groundwater potential [[23], [24], [25],^44^]. This part of the Tana sub-basin is a unique site where most of the productive wells have been located for the region's drinking, irrigation, and industrial demands. Unfortunately, groundwater pollution susceptibility is also high in this region [13]. Therefore, the Tana sub-basin's flat terrain is selected for the present study area which covers around 7,335 km^2^ of which Lake Tana takes 3000 km^2^ [7].

### 2.2 Groundwater sampling technique and laboratory analysis

Data including GPS (global positioning system) of GW points (hand-dug, deep wells, and spring water), LULC, lithological units, and soil maps were used to properly select the groundwater sampling sites [46]. Forty GW sampling points (Fig. 1) were selected purposively according to accessibility, availability of sampling points, land use land cover, lithological units, and areal distribution including deep (>60 m) and shallow (hand-dug, ≤ 30 m) wells including springs [7]. The last two letters (DW, HD, and SW) in the groundwater samples' name (Fig. 1) refer to deep well, hand-dug well and spring water, respectively while the first letters denote the site names’ acronyms. In each dry (January–March 2022) and wet (June–August 2022) study period, forty GW samples were obtained after 15–20 min of pumping deep and hand-dug wells including springs using half-litter polypropylene containers, which were cleansed with distilled water. Pumping (15–20 min) before sampling was done to avoid the water stored in the raising pipe besides wiping up contaminants at the mouth of faucets [47]. The major physicochemical GW quality parameters such as Na^+^, Mg^2+^, Ca^2+^, K^+^, Fe^2+^, SO_4_^2−^, Cl^−^, NO_3_^−^, HCO_3_^−^, CO_3_^2−^, PO_4_^3−^, pH, and EC were selected based on the earlier studies carried out in the study area [21,22].

In-situ assessment for the pH, EC, and TDS were estimated using a multi-parameter Palintest-800 device. Potassium sodium (Na^+^) and (K^+^) ions were estimated using a flame Atomic Absorption Spectroscopy (AAS) method. The remaining anions and cations considered in the present study were measured using a Multi-parameter photometer (Palintest-8000 photometer) following the guideline (https://sitefiles.camlab.co.uk/palintest%208000%20user%20manual%201167.pdf). Further descript ions of sampling, transporting, and laboratory analysis methods were explained in the previously published article by the present authors [7].

The laboratory results’ accuracy was tested by the ionic balance error method (IBE) (equation) since the accuracy may be reduced due to different sources of errors. In the dry and wet season laboratory analysis, less than ±10 % IBE was acceptable and considered for further analysis and interpretation [48,49]. However, it should be noted that all the laboratory results outside the acceptable range of IBE do not necessarily mean that the results are incorrect. Instead, the result indicates that some other unmeasured parameters significantly contribute to the ion balancing technique [50].

(1) $$\text{IBE}=\frac{\sum \text{Cations}-\sum \text{Anions}}{\sum \text{Cations}+\sum \text{Anions}}\times 100$$

where cations and anions are in milliequivalent per litter (meq/l).

### 2.3 Drinking water quality index (DWQI)

DWQI is a widely used and robust technique to assess the quality of drinking water [7,51]. DWQI is used to indicate the combined effect of various intensive and complex water quality data [[51], [52], [53]]. Three stages are required to calculate the DWQI using equations (2), (3), (4).

(2) $$W_{i}=\frac{w_{i}}{\sum_{i=1}^{n}\left(w_{i}\right)}$$

where *W*_*i*_ = each parameter's normalized weight; *n* = total number of parameters; *wi* is the weight given to each measured water quality parameter.

The weights from five to one were given for each hydro-geochemical parameter (Table 1) according to its significance for drinking and its influence on human health. A particular weight (w_i_) of 5 was allocated for NO_3_^−^ and EC (electrical conductivity); 4 for Fe^2+^ and SO_4_^2−^; 3 for pH, Cl^−^ and Na^+^; 2 for K^+^, Ca^2+^, and HCO_3_^−^, and 1 for Mg^2+^ following the recommendations of scholars [34,48,54].

**Table 1:** GW quality physicochemical parameters (in mg/L, unless specified), standards, and weights.

Parameters	WHO Standards [55]	ESA standards [56]	The weight given (wi)	The normalized weight (Wi)
pH (−)	6.5 to 8.5	6.5 to 8.5	3	0.09
EC (μS/cm)	1500	1500	5	0.15
Na^+^	200	200	3	0.09
K^+^	–	1.5	2	0.06
Ca^2+^	75	75	2	0.06
Mg^2+^	50	50	1	0.03
Fe^2+^	0.3	0.3	4	0.12
Cl^−^	250	250	3	0.09
SO_4_^2-^	250	250	4	0.12
NO_3_^−^	50	50	5	0.15

Secondly, *q*_*i*_ (quality rating) was computed using equation (3).

(3) $$q_{i}=\frac{C_{i}}{S_{i}}\times 100$$

where, *C*_*i*_ = the measured parameter's concentration, and *S*_*i*_ = the WHO's or Ethiopian standard's concentration (Table 1).

Finally, *DWQI* was determined using equation (4):

(4) $$\text{DWQI}=\sum_{i=1}^{n}W_{i}.q_{i}$$

After the *DWQI* was computed, GW quality classes such as excellent (DWQI< 50), good (50 ≤ DWQI<100), poor (100 ≤ DWQI<200), very poor (200 ≤ DWQI<300), and unsuitable (DWQI ≥ 300) were identified for drinking service [54].

### 2.4 Support vector machine (SVM)

SVM is an effective algorithm for handling the high-dimensional data of linear and non-linear relationships [37]. To investigate the spatial variation of GW quality, SVM was used since it performs best among the earlier commonly used geostatistical methods (inverse distance weighting (IDW) and different Kriging techniques) [7,37]. SVM also exhibits high performance from other machine learning algorithms comprising random subspace, artificial neural network, and additive regression [39,57]. Moreover, unlike other machine learning algorithms, SVM has a user-friendly Smart-Map QGIS plugin, which made it the best choice in this particular study.

Interpolation using SVM in the Smart-Map QGIS plugin tool needs DWQI values for each groundwater sample (B vector or observed values) and their corresponding GPS data (A matrix). Therefore, the longitude, latitude, and altitude data points of the forty GW samples were prepared in A matrix and added as features, which were computed using IDW. The B vector represents the observed values of DWQI to be interpolated based on a higher correlation with features (covariates). The robust spatial correlation global index of Moran [58] (equation (5)) is used to select covariates in Smart-Map SVM interpolation.

(5) $$I_{M}=\frac{\sum_{i=1}^{n}\left(\left(\sum_{j=1}^{n}w_{\text{ij}}\left(f_{j}-\bar{f}\right)\right).\left(\sum_{j=1}^{n}w_{\text{ij}}\left(g_{j}-\bar{g}\right)\right)\right)\;}{\sqrt{\left(\sum_{i=1}^{n}{\left(f_{i}-\bar{f}\right)}^{2}\right).\left(\sum_{i=1}^{n}{\left(g_{i}-\bar{g}\right)}^{2}\right)}}$$

where I_M_ is Moran's index; n is the number of in-situ samples (40 in the case of this study); f_i_ and f_j_ are the true (observed) values to be interpolated at i and j points; g_i_ and g_j_ are the selected covariates of observed values; f bar and g bar are the averages of f and y, respectively; and w_ij_ is the spatial weight with zero diagonal value of the matrix A element.

Details about the Smart-Map QGIS plugin tool and interpolation using SVM can be referred to Ref. [37].

## 3 Results

### 3.1 Groundwater physicochemical characteristics

The physicochemical characteristics and drinking suitability of the GW were assessed using pH, EC, TDS, anions (SO_4_^2−^, Cl^−^, NO_3_^−^, PO_4_^3−^, HCO_3_^−^, CO_3_^2−^), and cations (Na^+^, K^+^, Ca^2+^, M g^2+^, Fe^2+^) laboratory results as illustrated below.

#### 3.1.1 pH

The pH values varied from 6.00 to 7.80 and from 6.07 to 7.94 in dry and rainy periods, respectively. In the dry season, 77.5 % of samples were under the allowable limit, but the remaining 22.5 % were below 6.5 and thus, were acidic. The samples taken during the wet study period, however, had higher pH values and 80 % of them were alkaline and within the acceptable ranges for drinking. On the Durov diagram, the samples' average pH values were shown at the bottom (Fig. 4: (a) and (b)).

#### 3.1.2 EC and TDS

EC and TDS are the indicators of groundwater salinity and are the most frequently used parameters to assess and classify GW quality fitness for drinking [22,59,60]. If GW has EC value over the WHO standard, its quality for drinking purposes could be questionable. In the present study, EC and TDS were measured using a multi-meter micro 800 palintest. The result ranged from 105 to 2016 μS/cm in the dry and from 78.43 to 1754.00 μS/cm in the wet study period. The groundwater samples covering 80 % and 92.5 % collected in dry and wet periods, respectively, were within desirable standards for drinking water according to Refs. [55,56]. TDS was also found within the WHO permissible limit and the values were envisaged in the Gibbs diagram (Fig. 5, Fig. 6).

#### 3.1.3 Chloride (Cl^−^)

The sources of chloride in groundwater include rocks and geological weathering, domestic wastes, and agricultural inputs [61,62]. The chloride concentrations ranged from 0.00 to 186 mg/L with a 22.68 mg/L average, and from 0 to 195 mg/L with a 29.98 mg/L average were recorded in the wet and dry sampling periods, respectively. The higher value was recorded in the hand-dug well (named DELHD). All groundwater samples had increased chloride ion concentration, mainly in the shallow wells. Nonetheless, the overall chloride ion concentration in all sampled groundwater points fulfilled the suitability of drinking water since all were much below the permitted limit (250 mg/L).

#### 3.1.4 Nitrate (NO_3_^−^)

Nitrate is another essential parameter used to assess groundwater quality. The nitrate level in GW is usually trivial, but it could be increased due to different human activities, for instance, intensive agricultural practices, soil's organic matter decomposition, leaching from fertilizers, and domestic waste discharges [[63], [64], [65]]. Higher concentrations of nitrate above WHO standards (50 mg/L) [55] in drinking water poison the users' health. In addition, it causes several health problems, for example, methemoglobinemia in infants, diabetes, the risk for certain kinds of cancer, goitre, congenital disabilities, and hypertension [55,66,67].

**Fig. 2:**
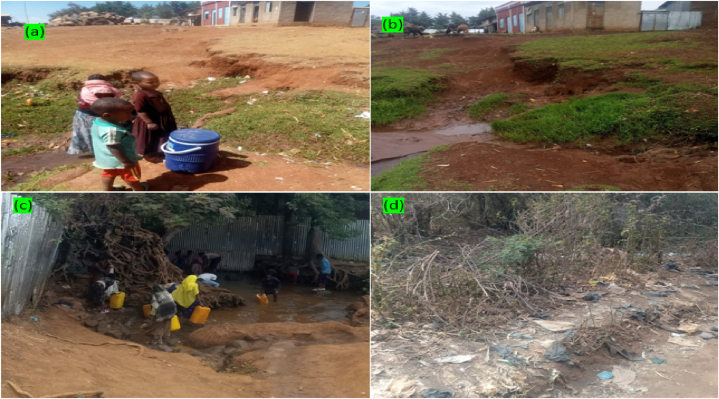
Photos taken during sample collection showing the sources of escalated nitrate concentration in AMTSW and MTSW spring water samples; (a) portrays storage of domestic and animal wastes around AMTSW during the dry period and (b) shows those pollutants entering the spring through runoff during wet season; (c) shows MTSW spring and (d) envisages open field toilet and other domestic wastes dump site just upstream of MTSW during dry season.

The nitrate levels in the dry GW samples ranged from 0.00 to 84.50 mg/L with a 16.46 mg/L average value, whereas in the wet GW samples varied from 1.88 to 94.00 mg/L with a 30.82 mg/L average value. In the dry study period, 90 % of the samples were under the allowable drinking water quality standard, but the remaining 10 % (comprised of MTSW, AMTSW, SERWMW, and HODGHD) were above the desired limit. The higher concentrations of nitrate ions were recorded in the rainy period than in the dry period and even, over the desired limit in the groundwater samples including WAHW, LHTHD, CHIMMW, ABRJHD, GURHD, and MITHD. In this wet season, only 75 % of the studied samples were in line with the acceptable nitrate drinking water quality standard. As observed during data collection, open field human defecation, incorrect domestic waste storage, and improper management of the wastes, in general, were especially, common in the cities (Fig. 2: (a) to (d)). Therefore, the increased amount of nitrate ions in GW during the wet study period may be due to precipitation and runoff-driven discharges from the nearby domestic and human-induced wastes and improper storage of animal manures (Fig. 2: (b) and (d)).

#### 3.1.5 Bicarbonate (HCO_3_^−^) and carbonate (CO_3_^2−^)

Bicarbonates and carbonates are generally derived from carbonate rocks, cations, and soluble salts. These anions lead to water hardness besides other calcium, magnesium chloride, and sulfate ions. A small body of studies suggested that excessive hard water drinking might cause heart problems and kidney stones [68,69]. The laboratory-measured concentration of bicarbonate, in the dry period, ranged from 9 to 410 mg/L with an average of 131.55 mg/L. In the study period of wet, on the other hand, the values extended from 10 to 460 mg/L with a 146.45 mg/L average. The carbonate ion levels fluctuated from 4.50 to 200 mg/L with a 62.15 mg/L average and from 5.23 to 230 mg/L with a 70.06 mg/L average.

The bicarbonate and carbonate concentrations were dominant and covered a high percentage of the overall GW samples (Table 2). Variations existed between the two study seasons. For example, in the wet season, increased bicarbonate concentrations were recorded. However, all samples of the bicarbonate concentrations were under the maximum allowable (500 mg/L) drinking water standard.

**Table 2:** Physicochemical statistical analysis (in mg/L, unless specified) in the dry and wet seasons.

Parameters	GW Quality Parameter Values							
	Dry season				Wet season			
	Minimum	Maximum	Mean	Standard deviation	Minimum	Maximum	Mean	Standard deviation
pH (−)	6.00	7.80	6.88	0.47	6.07	7.94	7.05	0.48
EC (μS/cm)	105.00	2016.00	1094.47	468.32	78.43	1754.00	921.82	380.16
Na^+^	1.60	317.45	54.85	68.16	0.55	316.25	52.40	67.36
K^+^	0.00	7.40	1.71	1.56	0.00	10.50	2.44	2.10
Ca^2+^	4.30	112.00	34.33	23.82	5.00	130.00	40.43	25.16
Mg^2+^	1.40	49.80	18.81	12.10	5.00	57.00	22.08	12.71
Fe^2+^	0.00	0.35	0.03	0.07	0.00	0.54	0.04	0.10
SO_4_^2+^	0.00	44.80	7.50	12.00	0.00	48.00	9.69	12.70
Cl^−^	0.00	186.00	22.68	37.65	0.00	195.00	29.98	39.83
NO_3_^−^	0.00	84.50	16.46	23.07	1.88	94.00	30.82	28.18
PO_4_^3-^	0.00	16.30	1.67	3.85	0.14	16.50	1.98	4.05
HCO_3_^−^	9.00	410.00	131.55	88.37	10.00	460.00	146.45	98.02
CO_3_^2-^	4.50	200.00	62.15	43.31	5.00	230.00	70.06	47.96

#### 3.1.6 Sulfate (SO_4_^2−^)

The sulfate existence in drinking water can lead to taste and odour [60,70]. Water having a higher level of sulfate than the maximum permissible limit by WHO standards could have a conspicuous taste and might hinder its use for drinking [55]. The sources of phosphate in the groundwater are primarily derived from weathering the naturally available sulfate-containing rocks (abundantly pyrite and gypsum) and intensive application of fertilizers [60,65]. The sulfate ion levels oscillated between 0 and 44.8 mg/L and 0–48 mg/L in the study seasons of dry and wet, respectively. In both study seasons, all GW samples were much less than the allowable limit (250 mg/L) of sulfate ion concentration**.**

#### 3. 1.8 Phosphate (PO_4_^−3^)

Phosphate can be available in groundwater from decomposing sedimentary rocks and natural minerals, fertilizers, industrial effluents, domestic wastes, and animal wastes [60,[71], [72], [73]]. WHO and Ethiopian Standard Agency (ESA) guidelines did not state the excess phosphate health effects or provide permissible limits/standards in drinking water. However, exceedingly high concentrations of phosphates can pose digestive problems, including overgrowth of small intestinal bacteria, nervous stomach, gastroesophageal reflux disease, and gallstones [74,75]. The laboratory analysis in this study indicated that phosphate ions extended from 0 to 6.30 mg/L with an average of 1.67 mg/L and from 0.14 to 16.5 mg/L with an average of 1.98 mg/L in the dry and wet seasons, respectively.

#### 3. 1.9 Calcium (Ca^2+^) and magnesium (Mg^2+^)

Appropriate Ca^2+^ and Mg^2+^ ions intake are vital for preventing hypertension, preeclampsia, bone health maintenance, body mass index increment, renal stones reduction, insulin resistance, and colorectal cancer [60,76]. However, the Ca^2+^ and Mg^2+^ increased levels in drinking water could be a source of diseases encompasses stomach disturbances, cardiovascular diseases, prenatal mortality, and different types of cancer [77]. The sources of these cations in GW may be limestone, sedimentary rocks, gypsum, dolomite, and other rocks [60,78]. The laboratory result of Ca^2+^ concentrations stretched from 4.3 to 112 mg/L with a 34.33 mg/L average value in the study season of dry and 5–130 mg/L with a 40.43 mg/L average in the study season of wet. The Mg^2+^ concentration, on the other hand, fluctuated from 1.40 to 49.80 mg/L with an 18.81 mg/L average value in the dry study period and 5–57 mg/L with a 22.08 mg/L value in the rainy study season. The higher values of calcium ion concentrations beyond the maximum tolerable boundary (75 mg/L) were recorded in the three sampled sites, namely CHEWMW, DELHD, and GURHD, in both study seasons. However, all the magnesium ion concentrations were far below the maximum allowable water quality standards for drinking during the study seasons. Escalated calcium and magnesium ion concentrations were observed in the wet period.

#### 3.1.9 Sodium (Na^+^) and potassium (K^+^)

Elevated sodium levels might cause blood pressure, heart disease, and kidney problems [60,79,80]. Adverse health risks from drinking water potassium consumption are rare since it is seldom found in drinking water and is rapidly excreted [81]. However, high potassium intake leads to high risk for infants, and old individuals, with problems such as renal insufficiency, kidney, heart disease, and coronary artery disease [81]. Nonetheless, unlike the Ethiopian standard agency (1.5 mg/L maximum limit), the WHO did not provide a standard of K^+^ in drinking water. The sodium ion concentrations extended from 1.60 to 317.45 mg/L with a 54.85 mg/L average in the dry and 0.55–316.25 mg/L with a 52.40 mg/L average in the rainy period. In both study seasons, a greater concentration of sodium ions was observed in SERWMW and ACHHD groundwater samples, which may indicate the presence of highly weathered quaternary geological formations or mineral soils on these sample sites. Potassium levels trenched from 0.00 to 7.40 mg/L with a 1.71 mg/L average in dry and 0–10.50 mg/L with a 2.44 mg/L mean in wet season. According to the [56] drinking water standard, 55 % and 30 % of the samples revealed K^+^ concentrations below the maximum drinking allowable boundary (1.5 mg/L) in dry and wet study periods, respectively.

#### 3.1.10 Iron (Fe^2+^)

In limited amounts, iron is among the essential elements required for a proper body [82,83]. Ingestion of high amounts of iron in drinking water can pose adverse health risks such as kidney, cardiovascular, liver, diabetes mellitus, hyperkeratosis, neurological disorders, respiratory, and other related health problems [[82], [83], [84]].

The concentration of ferrous ion (Fe^2+^) fluctuated between 0.00 and 0.54 mg/L in both two study seasons. In all samples, ferrous measured concentrations were within the desired limit except in the ZSHD sample. As observed during the sample collection, there was corrosion and cracked wellhead, especially in the wet season at the ZSHD hand pump, which may cause elevated ferrous concentration. Regarding the seasonal variation, higher values of ferrous ions were observed in the wet study period.

The statistical values of all the physicochemical parameters are tabulated in Table 2. It also depicts bicarbonate, carbonate, and sodium ions as the dominant parameters.

### 3.2 Hydrogeochemical facies and characteristics

The hydrogeochemical facies or water types of groundwater depend on water interaction with geological structure, mineralogy of aquifers, ion exchange, dissolution, and precipitation. Identifying the type of water has paramount importance for water resources management sustainably. The hydrogeochemical facies are determined by computing the percent contributions of each anion and cation and all ion concentrations in milliequivalent per litter (meq/L) for all the samples. The geochemical characteristics, water types and the geochemical process could best be envisaged by using the Piper and Durov diagrams in AquaChem software [34,35,70], and details about these diagrams including Gibbs diagram can be referred to Refs. [34,85].

**Fig. 3:**
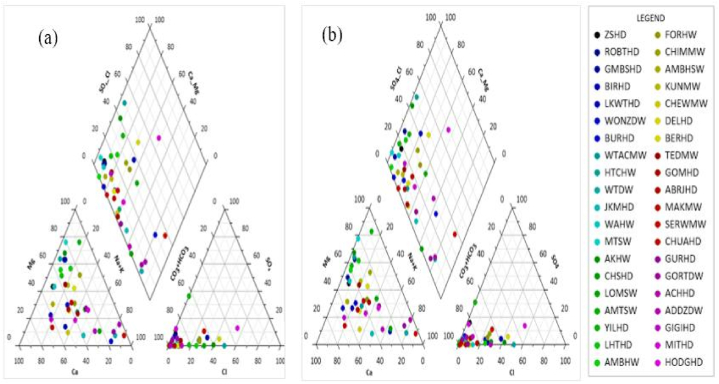
Piper diagram of the samples (listed in “LEGEND”) in the dry (a) and wet (b) seasons.

The cation triangle part of the Piper diagram (in Fig. 3: (a) and (b)) comprised the non-dominant magnesium, sodium, and calcium categories in the two study periods. The anion part of the piper diagram (Fig. 3: (a) and (b)), on the other side, was dominated by the bicarbonate category in the two seasons. The hydro-geochemical facies in the dry period (diamond part of Fig. 3: (a)) were Na–HCO_3_, Ca–HCO_3_, and Ca–Mg–Cl. The hydrogeochemical facies identified in the wet period were the same as those identified in the dry period and included Ca–Na–HCO_3_ (Fig. 3: (b)). Fig. 3 envisaged that the majority of GW samples were categorized under Ca–HCO_3_ in the two study periods.

**Fig. 4:**
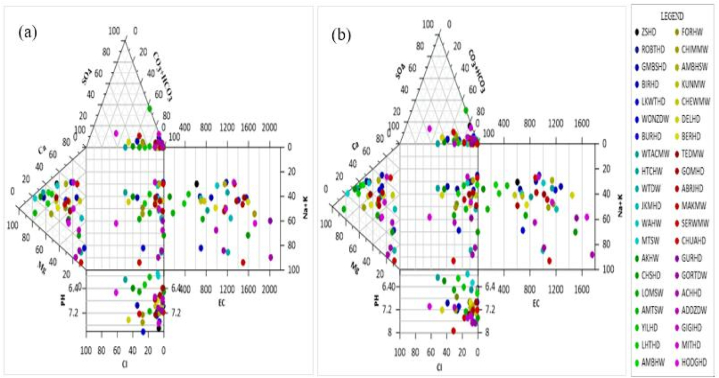
Durov diagram of the samples (listed in “LEGEND”) in the dry (a) and wet (b) seasons.

**Fig. 5:**
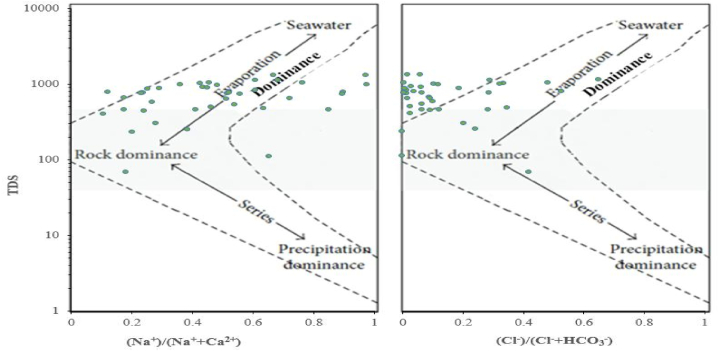
Gibbs diagram of the samples in the dry season.

**Fig. 6:**
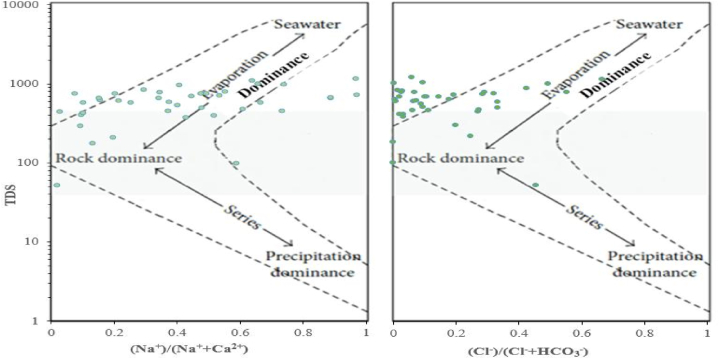
Gibbs diagram of the samples in the wet season.

The hydrogeochemical processes identified using Durov diagrams were mixing or simple dissolution, and reverse ion exchange (Fig. 4: (a) and (b)). The major hydro-geochemical process was a reverse ion exchange process as more groundwater samples were seen plotted at the right side (the reverse ion exchange zone) of the square part of the Durov diagram (Fig. 4: (a) and (b)).

The Gibbs diagram was also employed in the current study to identify mechanisms governing the hydrogeochemistry of the samples according to three distinct zones. These are rock dominance, precipitation dominance, and evaporation dominance [3,86]. The factors controlling the hydrogeochemical processes of cations and anions of GW systems in the dry and wet study periods were evaporation and rock dominance (Fig. 5, Fig. 6). Most of the hydro-geochemical analysis results were plotted in the evaporation-crystallization domain, and hence, the major mechanism governing hydro-geochemistry was evaporation in both study periods.

### 3.3 DWQI, seasonal and spatial variation

DWQI of the study region stretched from 17.63 to 82.96 and 19.85 to 86.65 in the dry and rainy seasons, respectively. It revealed that the overall GW quality falls into excellent and good water categories. Concerning the seasonal variation of the DWQI, higher values were observed in the wet period than in the dry period. In the dry study period, 80 % of groundwater samples presented excellent quality, but in the wet season, only 60 % were of excellent quality. However, the DWQI values of four deep groundwater samples (consisting of WONZDW, WTDW, GORTDW, and ADDZDW) did not show significant seasonal variation.

**Fig. 7:**
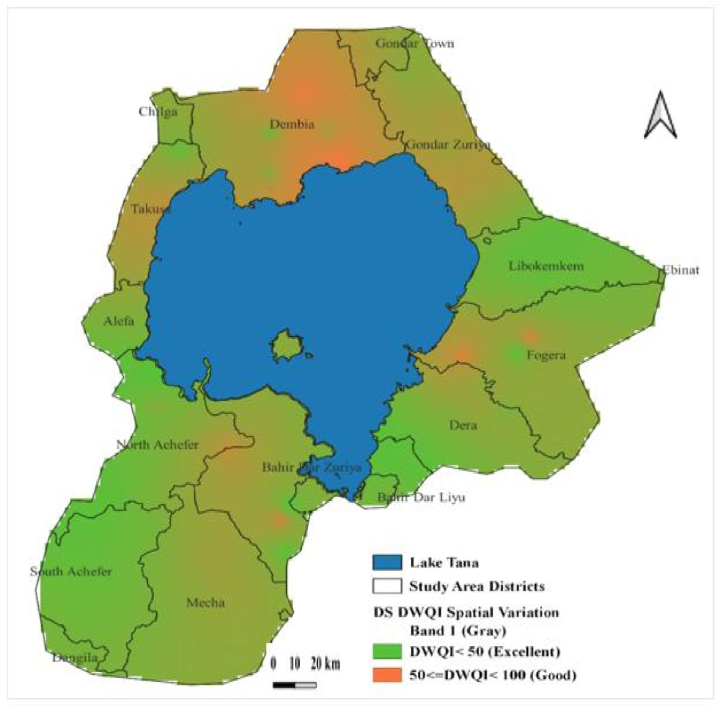
Groundwater quality spatial variation based on DWQI in the dry season (DS).

**Fig. 8:**
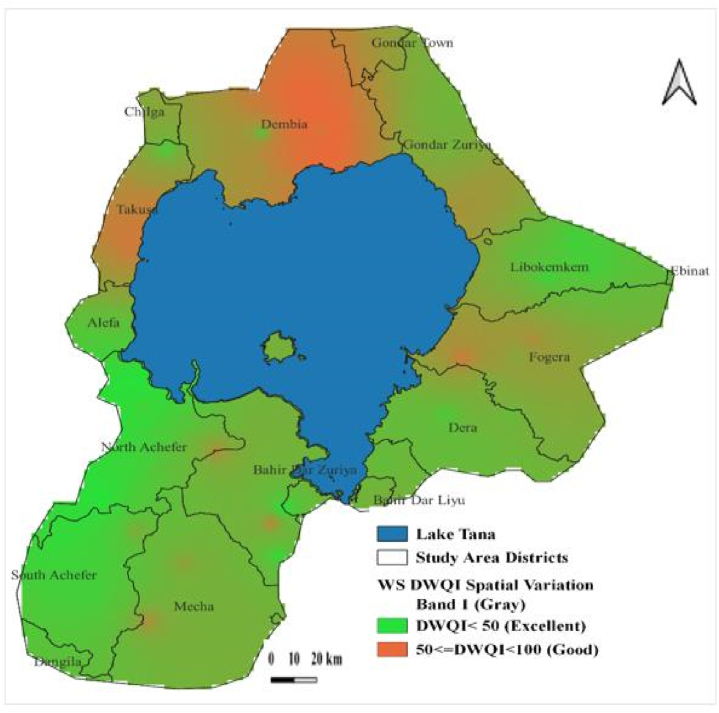
Groundwater quality spatial variation based on DWQI in the wet season (WS).

The main influencing parameters contributing to spatial groundwater quality variability were EC, Na^+^, Ca^2+^, K^+^ and NO_3_^−^. The reduced groundwater quality, classified here as good and highlighted in red (Fig. 7, Fig. 8), was observed in the southern, northern, and eastern directions of Lake Tana boundaries in both two study seasons. However, in the rainy season, the northern part of the study region showed more noticeable groundwater quality deterioration (Fig. 8). High-pollution risk zones have also been portrayed in a few areas with low groundwater quality shown in red (Fig. 7, Fig. 8).

## 4 Discussion

Groundwater quality physicochemical parameters’ laboratory estimation, hydrogeochemical facies, and the governing hydrogeochemical process were evaluated spatially and seasonally. The GW quality physicochemical parameters such as pH, EC, TDS, Cl^−^, NO_3_^−^, SO_4_^2−^, PO_4_^3−^, CO_3_^2−^ HCO_3_^−^, Ca^2+^, Mg^2+^, Na^+^, K^+^, and Fe^2+^, were estimated in laboratory analysis. The mean concentration values of each physicochemical parameter were comparable with previous studies conducted in the portion of the present study area [22] and in the depression of El Fayoum, Egypt [28].

The EC and TDS generally showed lower values in the dry than in the wet season. This might be mainly due to the increase in rainwater and concomitant ion dilutions in the wet season as supported by the previous works [87,88]. The nitrate levels during the rainy period showed an increment especially, in springs near settlements and shallow wells. This seemed because of pollutants from the nearby dumped wastes and agricultural fertilizers joining the groundwater through runoff and percolation. Studies [[89], [90], [91]] witnessed this idea. Chloride ion concentrations in all groundwater samples, especially in the shallow wells have lower values in dry than in wet study season. This may be because there was an increased leakage and infiltration of domestic and industrial effluents in the wet season due to precipitation [92].

The mean value of carbonate concentration did not show significant changes in the studied seasons, but bicarbonate's concentration may be due to weathering and dissolution of bicarbonate sources of aquifer materials (for instance, carbonate rocks) in the rainy season. This is in harmony with the earlier investigation [93]. The mean value of SO_4_^2^ levels of all the samples revealed miniature variations in the dry and wet seasons. However, relatively greater sulfate ion concentrations were recorded in the samples of wet study season (for example, SERWHD, ACHHD, and HODGHD), which may be linked with increased fertilizer applications and rain-derived infiltration to the sample points as witnessed by other previous works [64,65]. Higher concentrations of phosphate were detected in the wet than in the dry period, which can be related to the runoff and recharge of residues from farming areas and domestic wastes [60,94,95].

Escalated concentrations of Ca^2+^ and Mg^2+^ were observed in the wet study period. This could be connected to increased decipher of sedimentary rocks, gypsum, carbonate and other sources of calcium minerals in the wet period [60,96]. Unlike most of the other estimated physicochemical parameters, a lower sodium ion concentration was observed in the wet than in the dry period. This is because sufficient precipitation and recharge in the wet season diluted salts in groundwater and decreased salinity [97,98]. Higher concentrations of K^+^ were recorded in the wet than in the dry study period, which may be because of leaching from escalated fertilizers, pesticides, and animal wastes in the wet season [[99], [100], [101]]. Concentrations of K^+^ were by far or slightly lower than the Na^+^ concentrations of all samples in the two study seasons. This may be related to the potassium rocks’ lower weathering rate than sodium rocks since K^+^ could be detained by minerals of clay [99,102]. The ferrous levels showed an increment in the wet season but still were under the maximum allowable limit except for one GW sample concentration. This groundwater sample (labelled ZSHD) had a higher concentration especially in the wet season mainly due to a cracked wellhead and corroded galvanized hand pump. This rationale can be reinforced by the earlier research works [103,104].

The hydrogeochemical facies identified in the dry season were Ca–HCO_3_, Ca–Na–HCO_3_, and Na–HCO_3,_ whereas in the wet period were the aforementioned hydrogeochemical facies and Ca–Mg–Cl. The Ca–HCO_3_ took about 75 % of the samples in the two study seasons. A related study conducted in the whole Tana sub-basin also found the hydrogeochemical facies including Na–HCO_3,_ Ca–HCO_3_, and Ca–Na–HCO_3_ including others [18]. Other studies [47,105] conducted in the nearby area of the present study region also found Ca–HCO_3_ as the dominant water type. A very similar main hydrogeochemical facies (Ca–HCO_3_, Na–HCO3, and Ca/Mg–HCO3) were also obtained from the research work carried out in the Birr River watershed [11], a nearby area of the present study. The Ca–HCO_3_ revealed the recharging and freshwater quality [18,85,106]. As aforementioned above, one can understand that the water types could also vary from season to season. It was also evidenced by the earlier study [3]. Some of the GW samples that fell under the rock dominance zone of the Gibbs diagram were outside of the boomerang-shaped boundaries of the Gibbs diagram due to higher weight ratios and further extensions [107,108]. As could be understood from the Durov and Gibbs diagrams, the processes taking place in the hydrogeochemistry and controlling mechanisms of groundwater quality were reverse ion exchange, evaporation dominance, and rock weathering and dissolution dominance. According to the Gibbs diagram, most of the studied groundwater samples in both the two study seasons fell under the evaporation dominance zone. This was in line with the findings in the research conducted by Osta and his colleagues [27]. Other previous researchers [85] employed the Piper, Durov, and Gibbs diagram and found comparable results to this particular study. The recent work [18] also confirmed that the principal hydrogeochemical process controlling the groundwater of the Tana sub-basin is rock weathering and dissolution.

The groundwater pollution was more in the rainy period than in the dry period as identified using the DWQI approach. This might be attributed to the groundwater quality degradation, especially in the springs and shallow wells (which cover most of the groundwater samples) concomitant to the possible intrusion of polluted surface flood, and application of fertilizers [100,109]. DWQI results, generally, revealed good and excellent groundwater qualities, implying suitable for drinking purposes. An investigation carried out in a neighboring watershed [47] resulted in a similar outcome. Spatially, the reduced groundwater quality (good category) was mainly situated around Lake Tana's northern and southern directions in the two study periods. Especially, the good water quality class was conspicuously observed in the northern portion near Lake Tana. The presence of unsafe groundwater quality status was reported in the earlier study in the Dembia district (including the northern slice of the present study area) [22]. This evidence [22], confirmed that despite there being no unsuitable groundwater quality class for drinking in the present study, a relatively high groundwater quality degradation near Lake Tana was recorded.

This study might be beneficial to ascertaining a suitable well location, managing or confining the distribution of water pollutants for prevention, and suggesting remedial measures for groundwater contamination [[110], [111], [112]]. Furthermore, it has the potential to provide decisive figures of spatial and seasonal GW quality for drinking purposes. This investigation in general, may contribute to sustainable groundwater quality management, which is essential for unending holistic societal and economic developments of the studied region [113,114]. DWQI is a robust method to scrutinize large and complex water quality data into simple and understandable information to decision-makers but does not indicate the specific water quality situations and thus, be used with caution [33]. The current study also did not consider biological parameters, and hence, the subsequent research should focus on thorough groundwater quality assessment by including biological parameters to diminish health risks associated with drinking tainted water.

## 5 Conclusion

In the dry and wet study periods, Na^+^, Ca^2+^, Mg^2+^, K^+^, and Fe^2+^ were the dominating cations, whereas the controlling anions were, in decreasing order, HCO_3_^−^, CO_3_^2−^, Cl^−^ or NO_3_^−^, SO_4_^2−^, and PO_4_^3−^. The predominant water type found in the area, accounting for around 75 % of the GW samples, was calcium-bicarbonate (Ca–HCO3), also known as recharging water. The hydrogeochemical processes that regulate and manage groundwater in the study area were evaporation, reverse ion exchange, and the dissolution of sedimentary rocks and bicarbonate minerals. The dry season's groundwater quality was superior to the rainy season's, according to DWQI data. EC, Na^+^, Ca^2+^, K^+^ and NO_3_^−^ were the primary physicochemical characteristics that significantly influenced the seasonal and geographical quality fluctuations.

While outstanding quality was identified in other parts of the study territory far from the lake, a comparatively reduced groundwater quality class was detected in the northern portion of the region near Lake Tana. In the current work, the robust SVM is an excellent method for interpolating DWQI. The groundwater in the investigated area could be contaminated by surface elements, which might be harmful to human health. Therefore, to discourage surface pollutants, suitable waste disposal plans and waste development facilities should be implemented. This study in general, may provide decisive information on the spatial and seasonal quality groundwater resources for sustainable management and safe water supply in the studied region.

## Data availability statement

Data can be accessed in due request by the corresponding author.

## CRediT authorship contribution statement

**Kibru Gedam Berhanu:** Writing – original draft, Software, Investigation, Formal analysis, Conceptualization. **Tarun Kumar Lohani:** Writing – review & editing, Validation, Supervision, Project administration. **Samuel Dagalo Hatiye:** Writing – review & editing, Validation, Supervision, Data curation, Conceptualization, Visualization.

## Declaration of competing interest

The authors declare that there are no any competing interests.
