# Supplementary material for: Spatial and seasonal groundwater quality assessment for drinking suitability using index and machine learning approach
Source: Heliyon. 2024 Apr 27;10(9):e30362. doi: 10.1016/j.heliyon.2024.e30362 (PMC11089328; doi:10.1016/j.heliyon.2024.e30362)
Supplement: Multimedia component 1 [file mmc1.docx]

**Supplementary materials**


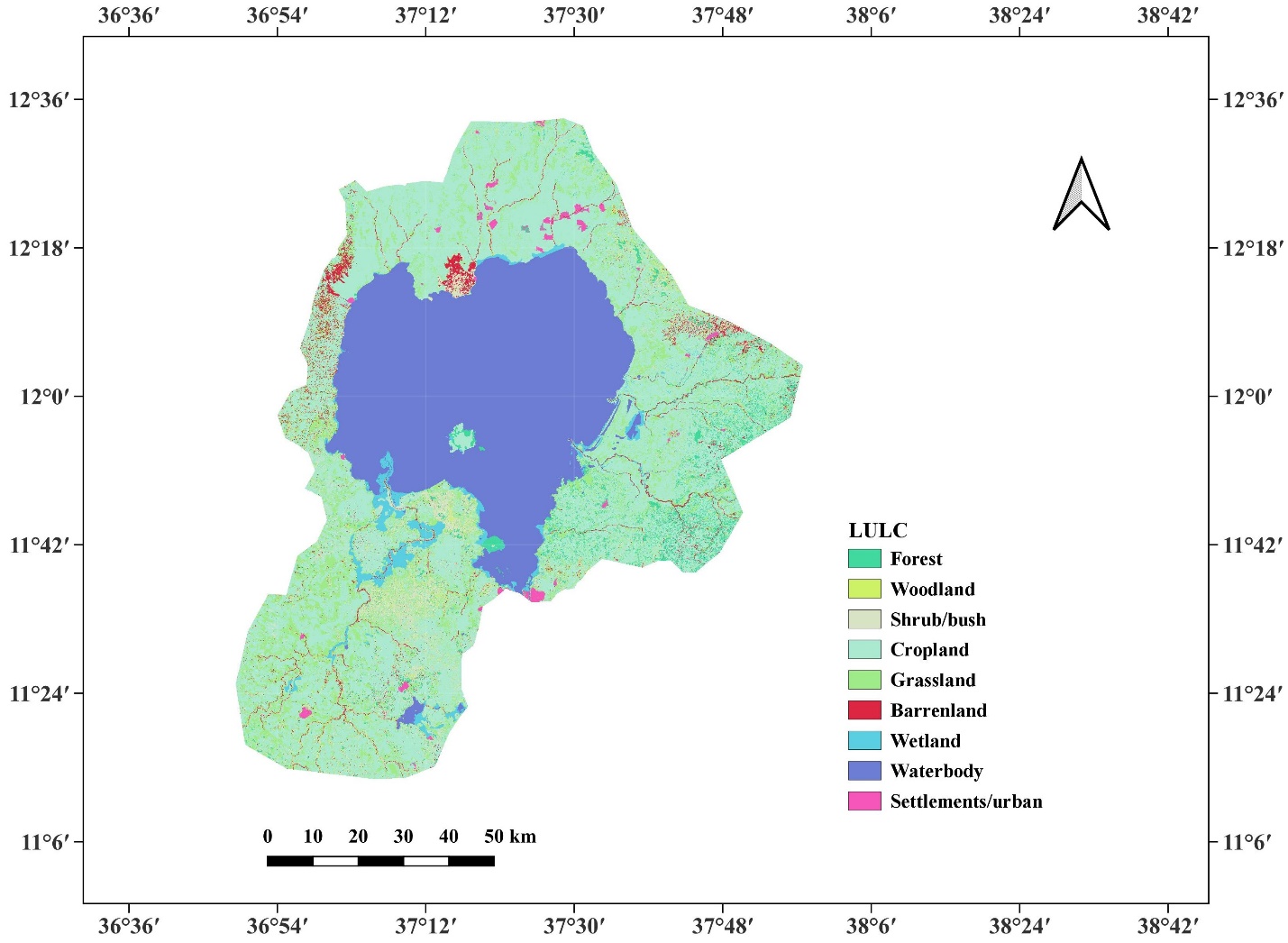


Figure S1: Land use/cover (LULC) map of the study area


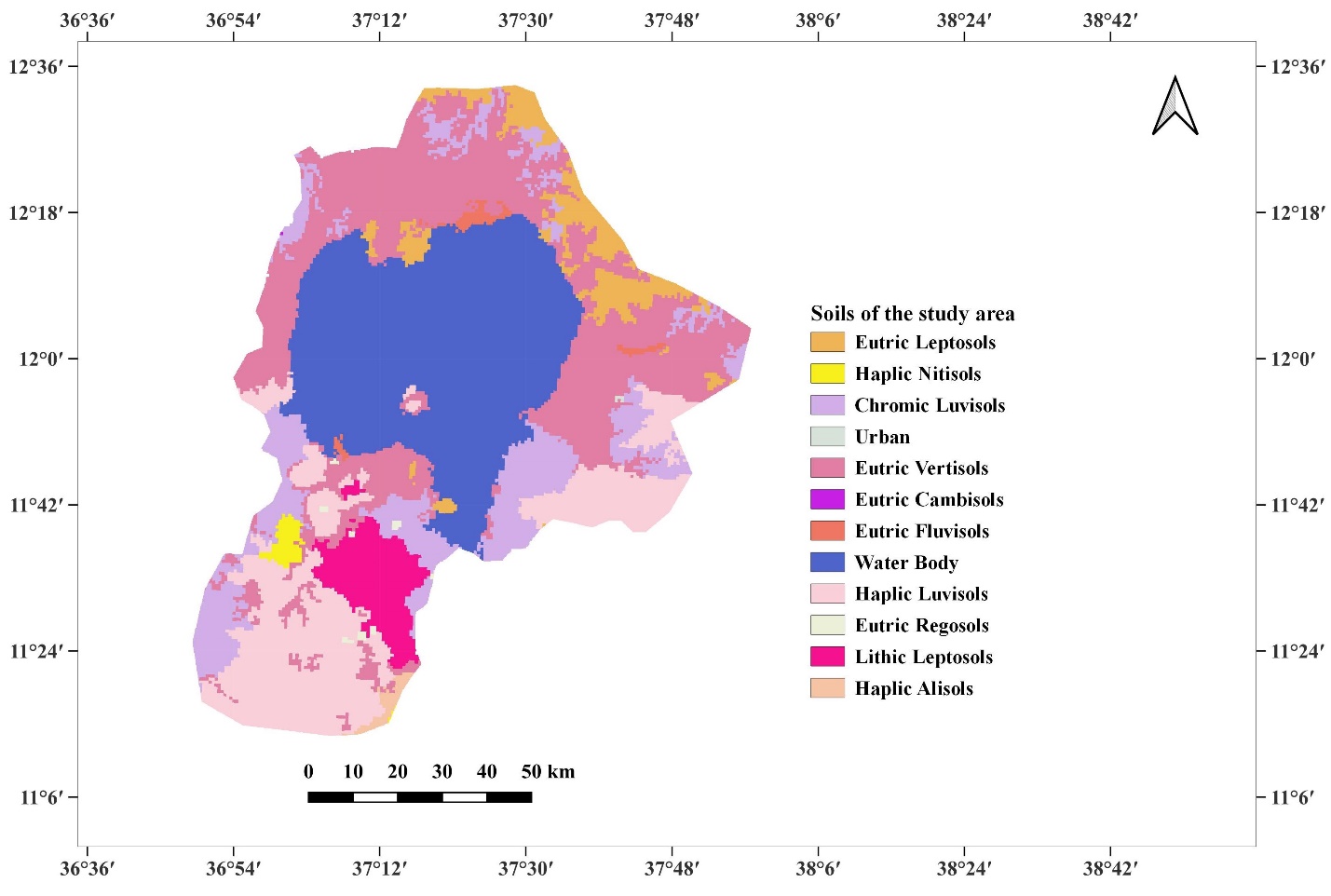


Figure S2: Soil types of the study area


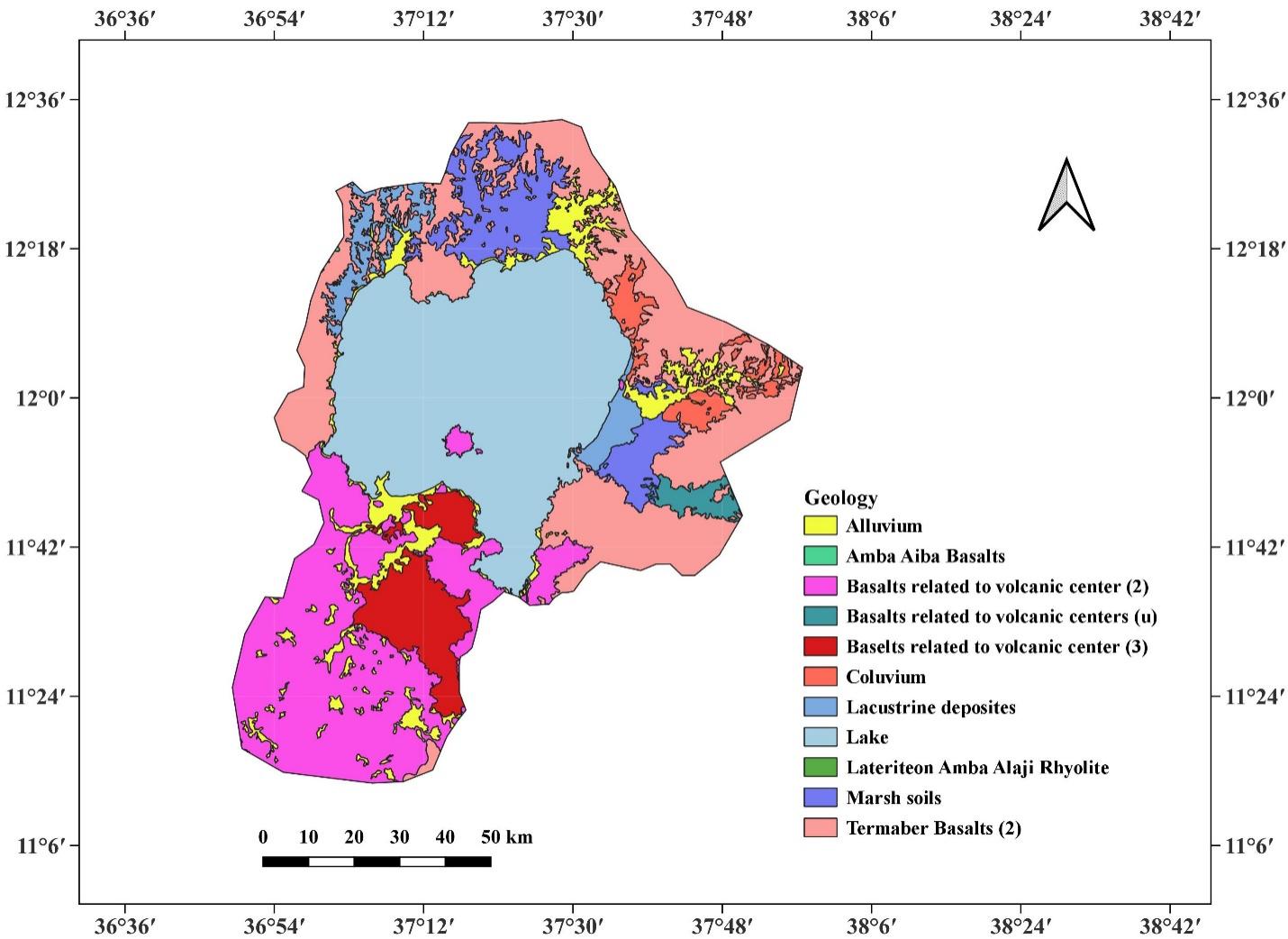


Figure S3: Geology (lithological units) of the study area
